# Socioeconomic Deprivation and Health Care Use in Patients Enrolled in SWOG Cancer Clinical Trials

**DOI:** 10.1001/jamanetworkopen.2024.4008

**Published:** 2024-03-28

**Authors:** Dawn L. Hershman, Riha Vaidya, Cathee Till, William Barlow, Mike LeBlanc, Scott Ramsey, Joseph M. Unger

**Affiliations:** 1 Columbia University, New York, New York; 2 SWOG Statistics and Data Management Center, Fred Hutchinson Cancer Research Center, Seattle, Washington

## Abstract

**Importance:**

Reducing acute care use is an important strategy for improving value. Patients with cancer are at risk for unplanned emergency department (ED) visits and hospital stays (HS). Clinical trial patients have homogeneous treatment; despite this, structural barriers to care may independently impact acute care use.

**Objective:**

To examine whether ED visits and HS within 12 months of trial enrollment are more common among Medicare enrollees who live in areas of socioeconomic deprivation or have Medicaid insurance.

**Design, Setting, and Participants:**

This cohort study included patients with cancer who were 65 years or older and treated in SWOG Cancer Research Network trials from 1999 to 2018 using data linked to Medicare claims. Data were collected from 1999 to 2019 and analyzed from 2022 to 2024.

**Main Outcomes and Measures:**

Outcomes were ED visits, HS, and costs in the first year following enrollment. Neighborhood socioeconomic deprivation was measured using patients’ zip code linked to the Area Deprivation Index (ADI), measured on a 0 to 100 scale for increasing deprivation and categorized into tertiles (T1 to T3). Type of insurance was classified as Medicare with or without commercial insurance vs dual Medicare and Medicaid. Demographic, clinical, and prognostic factors were captured from trial records. Multivariable regression was used, and the association of ADI and insurance with each outcome was considered separately.

**Results:**

In total, 3027 trial participants were analyzed. The median (range) age was 71 (65-98) years, 1280 (32.3%) were female, 221 (7.3%) were Black patients, 2717 (89.8%) were White patients, 90 (3.0%) had Medicare and Medicaid insurance, and 660 (22.3%) were in the areas of highest deprivation (ADI-T3). In all, 1094 patients (36.1%) had an ED visit and 983 patients (32.4%) had an HS. In multivariable generalized estimating equation, patients living in areas categorized as ADI-T3 were more likely to have an ED visit (OR, 1.34; 95% CI, 1.10-1.62; *P* = .004). A similar but nonsignificant pattern was observed for HS (OR, 1.36; 95% CI, 0.96-1.93; *P* = .08). Patients from areas with the highest deprivation had a 62% increase in risk of either an ED visit or HS (OR, 1.62; 95% CI, 1.25-2.09; *P* < .001). Patients with Medicare and Medicaid were 96% more likely to have an ED visit (OR, 1.96; 95% CI, 1.56-2.46; *P* < .001).

**Conclusions and Relevance:**

In this cohort of older patients enrolled in clinical trials, neighborhood deprivation and economic disadvantage were associated with an increase in ED visits and HS. Efforts are needed to ensure adequate resources to prevent unplanned use of acute care in socioeconomically vulnerable populations.

## Key Points

**Question:**

What is the association between socioeconomic factors and emergency department (ED) visits and hospital stays (HS) among individuals enrolled in Medicare who participate in cancer clinical trials?

**Findings:**

In this cohort study of 3027 patients aged 65 years or older who participated in a cancer clinical trial and had Medicare, 36.1% had an ED visit and 32.4% had an HS. Patients who lived in areas with the most socioeconomic deprivation had a 62% increase in risk of either an ED visit or HS; patients eligible for both Medicare and Medicaid were 96% more likely to have an ED visit.

**Meaning:**

These findings suggest that despite participation in cancer clinical trials, older patients living in areas with higher socioeconomic deprivation and those who are dual eligible for Medicaid and Medicare, which is a marker of economic disadvantage, have an increased risk of unplanned emergency care use.

## Teaser

This cohort study examines whether emergency department visits and hospital stays within 12 months of trial enrollment are more common among Medicare enrollees who live in areas of socioeconomic deprivation or have Medicaid insurance.

## Introduction

Patients with cancer from socioeconomically deprived areas (ie, geographical areas with a high proportion of people who are disadvantaged due to factors such as poverty, discrimination, or lack of access to basic necessities) have worse cancer outcomes.^1,2^ This has been attributed to the fact that socioeconomically deprived areas have limited access to screening and treatment services, and patients living in these areas tend to have more advanced disease at presentation.^3,4,5^ We have previously shown that even among patients enrolled in clinical trials with uniform treatment and after accounting for race, ethnicity, age, and insurance-related factors, patients from the most socioeconomically deprived areas had a greater risk of death and worse progression-free and cancer-specific survival. This suggests area-level deprivation and both cancer and noncancer outcomes may be associated independent of key patient-level sociodemographic factors.^5^

Population-based studies suggest that patients with cancer who are socioeconomically vulnerable have higher rates of emergency health care use.^6^ In a series evaluating 25 000 patients with advanced solid tumors from the California Cancer Registry, 71% of the patients were hospitalized in the year after diagnosis. Furthermore, the 67% of unplanned hospitalizations originated in the emergency department (ED). Race, ethnicity, insurance type, and socioeconomic status were all associated with hospital readmission rates. Reducing unplanned ED visits and hospital stays (HS) are an important strategy for improving the quality of care and reducing the cost of cancer care.

Studies have shown that the association between both area-level socioeconomic deprivation (ADI) and individual-level socioeconomic deprivation (as measured by insurance) and survival outcomes persist for patients treated in clinical trials.^5,7^ Less is known about the risk of noncancer outcomes and complications resulting in unplanned acute care, such as ED visits and HS, which may provide an opportunity for an intervention strategy.

## Methods

This cohort study follows the Strengthening the Reporting of Observational Studies in Epidemiology (STROBE) reporting guideline. Written informed consent for participants enrolled in each clinical protocol was previously obtained for all participants. Approval to conduct this research was obtained from the Institutional Review Board of Cancer Research and Biostatistics in Seattle, Washington.

We obtained data from the SWOG Cancer Research Network and included patients from clinical trials for 6 disease types (bladder, breast, colorectal, lung, prostate, and myeloma) (eTable 1 in Supplement 1). Trial records were linked to Medicare claims data according to social security number, sex, and date of birth. To be included, patients were required to be aged 65 years or older at time of enrollment and to have at least 12 months’ Medicare Parts A and B coverage with no concurrent HMO coverage.

Demographic variables, including age, sex, and self-reported race and ethnicity, were obtained at the time of enrollment. Race and ethnicity were assessed because they are known to be associated with increased health care use, as well as social determinants of health, and were collected prospectively by the clinical trials staff at each trial site. Potential differences in prognostic risk across the panel of different studies were accounted for using a study-specific prognostic risk score. For each study, we identified the key baseline clinical risk factors that were included as stratification variables in the trials. We then summed the number of adverse clinical risk factors, creating a composite risk score, standardized to a 0 to 100 scale, and split at the approximate median.^5^

Neighborhood socioeconomic deprivation was measured using patients’ residential zip code linked to the area deprivation index (ADI), which was measured on a 0 to 100 scale. Higher ADI denotes areas of higher deprivation. Patients missing zip code are not included.

HS were defined using the MedPAR file, by specifying NCH claims codes 60 to 64, 71, or 82, or, if claims code was missing, by specifying Skilled Nursing Facility indicator was not missing and was not N. HS with an admission date occurring within 1 year after registration were included. HS with different MedPAR ID were considered unique, even if dates of stay between 2 HS were overlapping.

ED visits were identified using 2 data sources: (1) outpatient revenue center data, using revenue center codes 0450-0459 and 0981; and (2) the MedPAR file, when the ED charge amount field was nonmissing and nonzero. As with HS, all ED visits occurring within 1 year after registration were included, with the potential of multiple observations per person.

To analyze health care costs as an outcome, claims cost data were compiled from MedPAR, Home Health Agency, outpatient, carrier, hospice, and durable medical equipment databases. Overall costs were examined, as well as separately by Medicare, beneficiary, and primary payers within the first 12 months after registration. Costs were inflated to 2021 US dollars based on the Personal Consumption Expenditure price index.

### Statistical Analysis

To assess the potential for bias, baseline characteristics were compared between those included in this analysis and those aged 65 years or older from the same studies who were not included due to HMO membership or lack of social security number. Generalized estimating equations (GEE) with a logit link were used to examine the binary health care use outcomes, accounting for clustering by cancer type. Analyses were conducted separately for HS (1 or more vs 0) or ED visit (1 or more vs 0), as well as a combined outcome, HS or ED visit (yes vs no to both). Two independent estimators were explored: ADI and insurance type. ADI was categorized into tertiles based on overall US distribution; the first tertile, representing areas with the least deprivation, was used as the referent category. Based on previous results in nontrial cancer patients, which showed differences in clinical outcomes between Medicare patients with vs without commercial insurance, we classified type of insurance at trial enrollment as Medicare alone, Medicare and commercial, or Medicare and Medicaid.^8^ As initial analyses showed similar use outcomes between patients with Medicare alone and Medicare and commercial insurance, for the primary insurance analysis, these groups were combined for increased power and to highlight the association of Medicaid insurance as an indicator of socioeconomic vulnerability. Both univariate and multivariate analyses were performed. Multivariable regression analyses included covariates for age (continuous), race (Black vs White vs other), study, and prognostic risk (above vs below the median). Given the limited number of patients with Medicaid insurance, no analysis of the interaction of insurance type and ADI was conducted. Instead, these variables were considered separately as area-level and individual-level measures of socioeconomic deprivation. We separately examined whether clustering at the study level rather than by cancer type meaningfully changed the findings.

Mean values of health use costs were found separately by insurance status, ADI tertile, and payer type. *P* values were calculated using linear mixed model regression with a log link under a gamma distribution for analyzing cost data, with cancer type as a random effect, adjusted for age, race, study, and baseline prognostic risk score. A 2-sided significance level *P* < .05 was chosen. The software package SAS version 9.4 (SAS Institute) was used for analyses. Data were collected from 1999 to 2019 (registrations ranged from 1999 to 2018, plus 1 year postregistration in 2019) and analyzed from 2022 to 2024.

## Results

In total, 3027 patients were analyzed. Median (IQR) age was 71 (65-98) years, 1280 (32.3%) were female, 221 (7.3%) were Black patients, and 2717 (89.8%) were White patients (Table 1). Compared with the patients not included from the same trials, the included patients were more likely to be White individuals; to be not Hispanic individuals; to be in breast, myeloma, or prostate cancer studies; to be registered in 2004 or later; and to have a lower prognostic risk score. Additionally, 913 patients (30.2%) had Medicare alone, 90 (3.0%) had Medicare and Medicaid insurance, and 2024 (66.9%) had Medicare and commercial. There were no differences in outcomes between patients with Medicare alone vs Medicare and commercial insurance (eTable 2 in Supplement 1). Thus, these groups were combined for our primary insurance analysis. Additionally, 1344 patients (45.4%) were in the lowest category of ADI (T1), and 660 (22.3%) were in the highest (T3). In all, 983 patients (32.4%) experienced HS, representing a total of 1805 HS, and 1094 patients (36.1%) had ED visits, representing a total of 2084 ED visits; and 1349 (44.6%) had HS or an ED visit.

**Table 1.** Patient Characteristics

Characteristic	Linked patients included in analysis (n = 3027)	Patients not linked and not included in analysis (n = 8636)	*P* value
Age, median (range), y	71 (65-98)	71 (65-97)	.91
Race			
Asian or Pacific Islander	46 (1.5)	172 (2.0)	<.001
Black	221 (7.3)	662 (7.7)	
American Indian or Alaska Native	12 (0.4)	38 (0.4)	
Unknown	31 (1.0)	882 (10.2)	
White	2717 (89.8)	6882 (79.7)	
Ethnicity			
Not Hispanic	2950 (97.5)	8236 (95.4)	<.001
Hispanic	77 (2.5)	400 (4.6)	
Sex			
Female	1280 (32.3)	3426 (39.7)	.01
Male	1747 (57.7)	5210 (60.3)	
Cancer type			
Bladder	131 (4.3)	585 (6.8)	<.001
Breast	1030 (34.0)	2303 (26.7)	
Colorectal	115 (3.8)	1343 (15.6)	
Lung	318 (10.5)	1078 (12.5)	
Multiple myeloma	118 (3.9)	274 (3.2)	
Prostate	1315 (43.4)	3053 (35.4)	
Time of initial registration			
Before 2004	935 (30.9)	3066 (35.5)	<.001
2004 or later	2092 (69.1)	5570 (64.5)	
Baseline prognostic risk score			
Low	1457 (48.5)	3726 (43.3)	<.001
High	1548 (51.5)	4874 (56.7)	
Missing, No.	22	36	
Insurance type			
Medicare alone	913 (30.2)	NA	NA
Medicare and Medicaid	90 (3.0)	NA	
Medicare and commercial	2024 (66.9)	NA	
ADI tertile			
T1 (most affluent)	1344 (45.4)	NA	NA
T2	954 (32.3)	NA	
T3 (most deprivation)	660 (22.3)	NA	
Missing, No.	69	NA	
No. of hospital stays			
None	2044 (67.5)	NA	NA
1	558 (18.4)	NA	
2	228 (7.5)	NA	
≥3	197 (6.5)	NA	
No. of ED visits			
None	1933 (63.9)	NA	NA
1	633 (20.9)	NA	
2	233 (7.7)	NA	
≥3	228 (7.5)	NA	

Abbreviations: ADI, Area Deprivation Index; ED, emergency department; NA, not applicable.

### Area Deprivation Index, Insurance, and Health Care Use

In multivariable GEE analysis, patients living in areas with the highest deprivation (ie, T3) were significantly more likely to experience an ED visit (OR, 1.34; 95% CI, 1.10-1.62; *P* = .004) (Table 2). A similar but nonsignificant association was seen with respect to HS (OR, 1.36; 95% CI, 0.96-1.93; *P* = .08). Overall, there was 62% increase in risk of either ED visit or HS for patients from areas with the highest deprivation (OR, 1.62; 95% CI, 1.25-2.09; *P* < .001).

**Table 2.** Socioeconomic Status and Risk of Hospital Stay and Emergency Department Visit

Outcome	Hospital stays or ED visit, No. (%)		Unadjusted		Adjusted^a^	
	No	Yes	OR (95% CI)	*P* value	OR (95% CI)	*P* value
**ADI tertiles^b^**						
Hospital stays						
T1 (most affluent)	961 (71.5)	383 (28.5)	1 [Reference]	NA	1 [Reference]	NA
T2	626 (65.6)	328 (34.4)	1.19 (1.01-1.39)	.04	1.15 (0.96-1.38)	.14
T3 (most deprivation)	408 (61.8)	252 (38.2)	1.38 (1.07-1.78)	.01	1.36 (0.96-1.93)	.08
ED visits						
T1 (most affluent)	914 (68.0)	430 (32.0)	1 [Reference]	NA	1 [Reference]	NA
T2	583 (61.1)	371 (38.9)	1.28 (1.14-1.45)	<.001	1.22 (1.07-1.39)	.003
T3 (most deprivation)	387 (58.6)	273 (41.4)	1.41 (1.20-1.66)	<.001	1.34 (1.10-1.62)	.004
Combined: hospital stay or ED visit						
T1 (most affluent)	809 (60.2)	535 (39.8)	1 [Reference]	NA	1 [Reference]	NA
T2	496 (52.0)	458 (48.0)	1.30 (1.11-1.53)	<.001	1.26 (1.08-1.47)	.003
T3 (most deprivation)	304 (46.1)	356 (53.9)	1.65 (1.35-2.02)	<.001	1.62 (1.25-2.09)	<.001
**Insurance type**						
Hospital stays						
Medicare alone or with private insurance	1939 (67.6)	929 (32.4)	1 [Reference]	NA	1 [Reference]	NA
Medicaid and Medicare	56 (62.2)	34 (37.8)	1.21 (0.86-1.72)	.27	1.19 (0.89-1.57)	.23
ED visits						
Medicare alone or with private insurance	1842 (64.2)	1026 (35.8)	1 [Reference]	NA	1 [Reference]	NA
Medicaid and Medicare	42 (46.7)	48 (53.3)	2.01 (1.53-2.64)	<.001	1.96 (1.56-2.46)	<.001
Combined: hospital stay or ED visit						
Medicare alone or with private insurance	1573 (54.8)	1295 (45.2)	1 [Reference]	NA	1 [Reference]	NA
Medicaid and Medicare	36 (40.0)	54 (60.0)	1.80 (1.24-2.59)	.002	1.73 (1.33-2.25)	<.001

Abbreviations: ADI, Area Deprivation Index; ED, emergency department; NA, not applicable; OR, odds ratio.

^a^ Odds ratios and *P* values calculated using generalized estimating equations with a logit link, accounting for clustering by cancer type, and adjusted for age (continuous), race (Black individuals compared with White individuals compared with other, including American Indian or Alaskan Native, Asian or Pacific Islander, and all other groups), study, and prognostic risk score.

^b^ ADI tertiles are defined as: T1, 46.9 or less; T2, 47.0 to 68.9; T3, 69.0 or more.

In adjusted analyses, patients with Medicare and Medicaid insurance were more likely to have ED visit in the first year (OR, 1.96; 95% CI, 1.56-2.46; *P* < .001; Table 2). In contrast, no increased risk of HS for Medicare and Medicaid patients was observed. The findings were similar when clustering was at the study level rather than cancer level (eTable 3 in Supplement 1).

### Costs of Health Care Use

Patients from areas with the highest deprivation had greater total mean (SD) costs than those from the most affluent areas ($46 070.55 [$46 769.98] vs $40 547.39 [$42 755.70]; *P* < .001). Findings were similarly discrepant for costs paid by Medicare and costs paid by the patient (Table 3). In contrast, mean total, Medicare, and patient costs trended higher for patients with Medicaid and Medicare compared with those with Medicare and commercial insurance, the differences were not statistically significant.

**Table 3.** Socioeconomic Status and Costs of Health Care Use in First 12 Months

Socioeconomic measure	Total costs		Costs paid by Medicare		Costs paid by patient	
	Observed cost, mean (SD), $	*P* value^a^	Observed cost, mean (SD), $	*P* value^a^	Observed cost, mean (SD), $	*P* value^a^
ADI^b^						
T1 (most affluent)	40 547.39 (42 755.70)	NA	32 165.42 (35 746.99)	NA	7423.78 (7855.99)	NA
T2	45 893.79 (43 130.78)	.05	36 103.54 (35 976.21)	.09	8767.62 (9183.40)	.27
T3 (most deprived)	46 070.55 (46 769.98)	<.001	36 535.14 (39 655.34)	<.001	8495.21 (8400.91)	<.001
Insurance type						
Medicare alone or with private insurance	42 982.69 (43 358.69)	NA	33 951.63 (36 283.88)	NA	8023.75 (8415.39)	NA
Medicaid and Medicare	53 634.75 (51 247.03)	.74	44 194.98 (44 360.63)	.92	9439.78 (7626.56)	.71

Abbreviations: ADI, Area Deprivation Index; NA, not applicable.

^a^ *P* values calculated using linear mixed model regression with log link and gamma family, with cancer type as random effects, adjusted for age (continuous), race (Black vs White vs other), study, and prognostic risk score.

^b^ ADI tertiles are defined as: T1, 47.0 or less; T2, 47.1 to less than 69.0; T3, 69.0 or more.

## Discussion

In this cohort study of older patients enrolled in clinical trials, ED visits and HS were frequent within the first year following clinical trial enrollment. Both neighborhood deprivation and economic disadvantage as measured by the presence of Medicaid insurance were associated with increased ED visits and HS within the first 12 months among patients with Medicare. Therefore, despite participation in cancer clinical trials, older patients living with higher social needs have an increased risk of unplanned emergency care use. Moreover, the observed association increased as area level deprivation progressed from most affluent to most socioeconomically vulnerable, improving confidence in the validity of the findings.

The rising cost of cancer care is a major public health issue, and with newer, more targeted therapies, the costs are likely to increase. Decreasing unplanned hospitalizations provides an opportunity to decrease costs. Ironically, we found that the total care costs, those paid by Medicare and those paid by the patient, were lowest among patients who lived in the most affluent areas. Previous reports suggest that more than two-thirds of unavoidable hospitalizations are the result of cancer-related symptoms. One proven solution to reduce unplanned hospitalizations among cancer patients is active symptom monitoring.^9^ It is known that active symptom monitoring of patients undergoing chemotherapy has reduced health care use, improved quality of life, and increased survival.^10,11^ In the community setting symptom monitoring with electronic patient reported outcomes decreased hospitalizations from 32% to 20%, emergency visits from 42% to 38% and reduced the total cost of care by an average of $1146 per member per month.^12^

Several prior studies have reported an association between socioeconomic status and risk of hospital readmissions. A machine learning approach based on 13 000 cancer patients found that 30-day readmission was associated with neighborhood income, wealth index, crime index, home values, and comorbidity index.^13^ Similarly, a study using the California Cancer Registry linked to inpatient discharge data found that rehospitalization was associated with Black race, Hispanic ethnicity, public insurance or no insurance, and lower socioeconomic status. Fewer studies have evaluated nonclinical factors associated with initial unplanned hospitalization.^14^

### Strengths and Limitations

There are several strengths to our study. Participants were prospectively enrolled and baseline data was collected on all patients. For each study, patients were required to adhere to uniform protocol-specific therapy. All of the patients in this cohort were treated uniformly. Uniform access to protocol therapy also limits the confounding influence of initial access to cancer care.

This study has limitations. Patients were required to be enrolled in Medicare to be included in this study, thus all analyzed patients were older than age 65 years. Given that older patients are often underrepresented in clinical trials, selection bias may limit the generalizability of the results. All SWOG Cancer Research Network studies mandated a Zubrod score of 0 to 2, specifying that patients needed to be at least ambulatory and capable of self-care, as part of the inclusion criteria. Thus, patients with severe complications may not have been captured, which could also limit the generalizability of our results. Additionally, the reason for the hospitalization or ED visit was not available, so it was not possible to know how many could have been avoided. Finally, because the multilevel modeling strategy aims to identify aggregate patterns across a diverse set of cancer types and trials, results at the individual cancer categories and trial levels are not readily interpretable.

## Conclusions

In this cohort study of elderly patients enrolled in clinical trials, neighborhood deprivation and economic disadvantage were associated with an increase in ED visits and HS. As a result, where a patient receives their care can account for disparities in outcome, even among clinical trials participants. Identifying patients with the highest risk may be an helpful strategy for targeted interventions. Policies to mitigate socioeconomic differences in cancer outcomes should emphasize access to cancer care services during and beyond initial therapy. Substantial efforts to increase diversity in clinical trials participation are under way.^15^ Efforts are needed to ensure adequate resources to prevent unplanned use of acute care in vulnerable populations.

## Notes

Supplement 1. **eTable 1.** Clinical Trial Characteristics and Prognostic Risk Score **eTable 2.** Socioeconomic Status and Risk of Hospital Stay and Emergency Room Visit With Insurance Broken Out by Medicare Alone, Medicare+Private, Medicaid+Medicare **eTable 3.** Socioeconomic Status and Risk of Hospital Stay and Emergency Room Visit With Clustering by Study ID Rather Than Cancer Type

Supplement 2.
Data Sharing Statement
