# Supplementary material for: Socioeconomic Deprivation and Health Care Use in Patients Enrolled in SWOG Cancer Clinical Trials
Source: JAMA Netw Open. 2024 Mar 28;7(3):e244008. doi: 10.1001/jamanetworkopen.2024.4008 (PMC10979311; doi:10.1001/jamanetworkopen.2024.4008)
Supplement: Supplement 1. — eTable 1. Clinical Trial Characteristics and Prognostic Risk Score eTable 2. Socioeconomic Status and Risk of Hospital Stay and Emergency Room Visit With Insurance Broken Out by Medicare Alone, Medicare+Private, Medicaid+Medicare eTable 3. Socioeconomic Status and Risk of Hospital Stay and Emergency Room Visit With Clustering by Study ID Rather Than Cancer Type [file jamanetwopen-e244008-s001.pdf]

Supplemental Online Content

Hershman DL, Vaidya R, Till C, et al. Socioeconomic deprivation and health care use in patients enrolled in SWOG cancer clinical trials. *JAMA Netw Open*. 2024;7(3):e244008. doi:10.1001/jamanetworkopen.2024.4008

eTable 1. Clinical Trial Characteristics and Prognostic Risk Score

eTable 2. Socioeconomic Status and Risk of Hospital Stay and Emergency Room Visit With Insurance Broken Out by Medicare Alone, Medicare+Private, Medicaid+Medicare

eTable 3. Socioeconomic Status and Risk of Hospital Stay and Emergency Room Visit With Clustering by Study ID Rather Than Cancer Type

This supplemental material has been provided by the authors to give readers additional information about their work.

© 2024 Hershman DL et al. *JAMA Network Open.*

eTable 1: Clinical Trial Characteristics and Prognostic Risk Score

Study	Risk Factor	Risk Score	"High-Risk" Sum[1]	Total Registered	Number Included in Analysis	N (%) "High-Risk" in this Analysis
Bladder Studies						
S0337	Recurrence (vs 1st occurrence)	1	≥1	416	74	44 (59%)
	2+ tumors (vs 1 tumor)	1				
S1011	Performance status: 2 (vs 0/1)	1	>0	658	25	13 (52%)
	Clinical Stage: III or IIIA (vs II)	1				
S9809	Recurrence (vs 1st occurrence)	1	=1	114	32	21 (66%)
Breast Studies						
S0012	Disease status: inflammatory	1	=1	399	25	13 (52%)
S0221	HER-, ER+ &/or PGR+	0	≥2	3294	151	43 (31%)
	HER+	1				
	HER-, ER-, and PGR-	2				
	Number of positive nodes >=4	1				
S0226	Prior adjuvant tamoxifen therapy: yes (vs no)	1	=1	707	178	62 (36%)
S0307	Number of positive nodes >=4	1	≥1	6097	441	67 (15%)
	Tumor size ≥5cm	1				
S0500	HER2-	1	=2	624	55	41 (75%)
	Measurable disease (vs bone-only disease)	1				
S1007	Recurrence Score 14-25 (vs 0-13)	1	=2	5083	136	71 (52%)
	Post-menopausal	1				
S1207	≥ 1 positive lymph node after neoadjuvant chemotherapy.[1]	1	=1	1939	44	10 (23%)
Colorectal Studies						
S0600	Performance status: 1+ (vs 0)	1	≥2	72	7	5 (71%)
	Oxaliplatin: discontinued (vs not discontinued)	1				
	Bevacizumab dose 14-42 days ago (vs 43+ days)	1				
S9304	Nodal status: 4+ (vs 0-3)	1	>0	1917	64	22 (34%)
	T-stage: T4b (vs T1-2 or T3)	1				
S9415	T-stage: T4 (vs T1/T2/T3)	1	>0	1135	32	11 (34%)
	N-stage: N2-3 (vs N0/N1)	1				
S9420	Performance status: 2 (vs 0/1)	1	=1	730	12	1 (8%)
Lung Studies						
S0003	Weight loss: ≥5% (vs <5%)	1	≥1	397	41	38 (93%)
	Stage: IV (vs IIIB)	1				
	LDH>IULN (vs LDH≤IULN)	1				
S0023	T-stage: T3/T4 (vs T1/T2)	1	≥2	620	74	38 (52%)
	N-stage: N3 (vs N0/N1/N2)	1				
	Squamous histologic/cytologic subtype (vs nonsquamous)	1				
S0124	LDH: >IULN (vs ≤IULN)	1	≥2	671	61	34 (56%)

© 2024 Hershman DL et al. *JAMA Network Open.*

	Metastatic sites: multiple (vs single)	1				
	Weight loss: ≥5% (vs <5%)	1				
S0819	Bevacizumab inappropriate (vs appropriate)	1	≥2	1333	80	35 (44%)
	Smoking history: current (vs former/never)	1				
	M-stage: M1b (vs M1a)	1				
S9900	Clinical stage: IIB/IIIA (vs IB/IIA)	1	>0	354	62	10 (16%)
Multiple Myeloma						
S0232	Performance status: 2-3 (vs 0/1)	1	≥3	198	56	17 (30%)
	ISS Stage I	1				
	ISS Stage III	2				
	ISS Stage III	3				
S0777	Intent to transplant at progression: N (vs Y)	1	≥2	525	51	42 (82%)
	ISS Stage I	1				
	ISS Stage III	2				
	ISS Stage III	3				
S9321	Performance status: ≥2 (vs 0/1)	1	>0	902	11	3 (27%)
	Baseline serum beta-2 microglobulin: ≥6 ug/mL (vs <6 ug/mL)	1				
Prostate Studies						
S0421	Type of progression: PSA only (vs no measurable/evaluable)	1	≥2	1038	244	137 (56%)
	Bisphosphonate use: yes (vs no)	1				
	BPI worst pain: ≥4 (vs <4)	1				
	Extraskeletal metastases: yes (vs no)	1				
S1216	Disease severity: extensive (vs minimal)	1	≥1	1313	79	60 (76%)
	Perforance status: 2-3 (vs 0-1)	1				
	Pre-registration treatment status: late vs. early induction	1				
S9346	Bone Metastases: Yes for any (vs No/Unknown for all)	1	>1	3040	599	506 (85%)
	Bone Pain: Yes (vs No/Unknown)	1				
S9916	Type of progression: measurable/evaluable disease (vs PSA only)	1	>1	770	215	181 (84%)
	Bone pain: grade ≥2 (vs grade <2)	1				
	Performance status: 2/3 (vs 0/1)	1				
S9921	Pathologic stage of disease: N1 (vs organ confined but N0 or not organ confined but N0)	1	>0	983	178	23 (13%)
	Gleason sum: >7 (vs ≤7)	1				

[1] Low risk included the complementary categories: 1) Node-negative and Oncotype DX® Recurrence Score > 25 or a MammaPrint® assay in the high-risk category in the primary tumor, and a tumor measuring ≥ 2 cm in greatest diameter treated with adjuvant therapy, or 2) 1-3 positive lymph nodes and Oncotype DX® Recurrence Score > 25 or a MammaPrint® assay in the high-risk category or Grade III disease treated with adjuvant therapy, or 3) ≥ 4 positive lymph nodes treated with adjuvant therapy.

© 2024 Hershman DL et al. *JAMA Network Open.*

eTable 2: Socioeconomic Status and Risk of Hospital Stay and Emergency Room Visit with Insurance broken out by Medicare Alone, Medicare+Private, Medicaid+Medicare

	Hospital Stay or ER Visit		Unadjusted		Adjusted[1]	
	No, N (%)	Yes, N (%)	OR (95% CI)	p-value	OR (95% CI)	p-value
**Insurance type**						
Hospital Stays						
Medicaid+Medicare	56 (62.2%)	34 (37.8%)	Ref		Ref	
Medicare Alone	589 (66.0%)	304 (34.0%)	0.88 (0.62-1.25)	0.48	0.90 (0.68-1.18)	0.45
Medicare+Private	1350 (68.4%)	625 (31.6%)	0.80 (0.56-1.14)	0.21	0.81 (0.60-1.10)	0.17
Emergency Room Visits						
Medicaid+Medicare	42 (46.7%)	48 (53.3%)	Ref		Ref	
Medicare Alone	554 (62.0%)	339 (38.0%)	0.54 (0.41-0.73)	<0.001	0.54 (0.42-0.70)	<0.001
Medicare+Private	1288 (65.2%)	687 (34.8%)	0.48 (0.36-0.63)	<0.001	0.50 (0.40-0.62)	<0.001
Combined: Hospital Stay or Emergency Room Visit						
Medicaid+Medicare	36 (40.0%)	54 (60.0%)	Ref		Ref	
Medicare Alone	468 (52.4%)	425 (47.6%)	0.61 (0.42-0.89)	0.010	0.62 (0.46-0.83)	0.001
Medicare+Private	1105 (55.9%)	870 (44.1%)	0.53 (0.37-0.78)	0.001	0.56 (0.43-0.73)	<0.001

[1] Odds ratios and p-values calculated using generalized estimating equations with a logit link, accounting for clustering by cancer type, and adjusted for age (continuous), race (Black vs non-Black), study, and prognostic risk score

© 2024 Hershman DL et al. *JAMA Network Open.*

eTable 3: Socioeconomic Status and Risk of Hospital Stay and Emergency Room Visit with Clustering by study ID rather than cancer type

	Hospital Stay or ER Visit		Unadjusted		Adjusted[1]	
	No, N (%)	Yes, N (%)	OR (95% CI)	p-value	OR (95% CI)	p-value
**ADI Tertiles**						
Hospital Stays						
T1 (most affluent)	961 (71.5%)	383 (28.5%)	Ref		Ref	
T2	626 (65.6%)	328 (34.4%)	1.11 (0.94-1.32)	0.23	1.15 (0.92-1.43)	0.23
T3 (most deprived)	408 (61.8%)	252 (38.2%)	1.26 (0.96-1.66)	0.10	1.36 (0.98-1.89)	0.06
Emergency Room Visits						
T1 (most affluent)	914 (68.0%)	430 (32.0%)	Ref		Ref	
T2	583 (61.1%)	371 (38.9%)	1.22 (1.02-1.44)	0.03	1.22 (1.01-1.48)	0.04
T3 (most deprived)	387 (58.6%)	273 (41.4%)	1.32 (1.15-1.52)	<0.001	1.34 (1.13-1.58)	<0.001
Combined: Hospital Stay or Emergency Room Visit						
T1 (most affluent)	809 (60.2%)	535 (39.8%)	Ref		Ref	
T2	496 (52.0%)	458 (48.0%)	1.23 (0.98-1.53)	0.07	1.26 (0.99-1.62)	0.06
T3 (most deprived)	304 (46.1%)	356 (53.9%)	1.51 (1.26-1.83)	<0.001	1.62 (1.32-1.99)	<0.001
**Insurance type**						
Hospital Stays						
Medicare Alone or +Private	1939 (67.6%)	929 (32.4%)	Ref		Ref	
Medicaid+Medicare	56 (62.2%)	34 (37.8%)	1.11 (0.78-1.57)	0.57	1.19 (0.79-1.78)	0.41
Emergency Room Visits						
Medicare Alone or +Private	1842 (64.2%)	1026 (35.8%)	Ref		Ref	
Medicaid+Medicare	42 (46.7%)	48 (53.3%)	1.86 (1.36-2.53)	<0.001	1.96 (1.45-2.64)	<0.001
Combined: Hospital Stay or Emergency Room Visit						
Medicare Alone or +Private	1573 (54.8%)	1295 (45.2%)	Ref		Ref	
Medicaid+Medicare	36 (40.0%)	54 (60.0%)	1.61 (1.08-2.39)	0.02	1.73 (1.20-2.49)	0.003

[1] Odds ratios and p-values calculated using generalized estimating equations with a logit link, accounting for clustering by study ID, and adjusted for age (continuous), race (Black vs non-Black), study, and prognostic risk score

ADI Tertiles are defined as: T1: ≤46.9, T2: 47.1 to <69.0, T3: ≥69.0

© 2024 Hershman DL et al. *JAMA Network Open.*
