# Supplementary material for: Socioeconomic Deprivation and Health Care Use in Patients Enrolled in SWOG Cancer Clinical Trials
Source: JAMA Netw Open. 2024 Mar 28;7(3):e244008. doi: 10.1001/jamanetworkopen.2024.4008 (PMC10979311; doi:10.1001/jamanetworkopen.2024.4008)
Supplement: Supplement 2. — Data Sharing Statement [file jamanetwopen-e244008-s002.pdf]

Data Sharing Statement

Data
Data available: Yes
Data types: Deidentified participant data
How to access data: Email PI
When available: With publication

Supporting Documents
Document types: None

Additional Information
Who can access the data: researchers whose proposed use of the data has been approved
Types of analyses: for a specified purpose
Mechanisms of data availability: with a signed data access agreement
Any additional restrictions: NA
